# Cost Analysis of Health Examination Screening Program for Ischemic Heart Disease in Active-Duty Military Personnel in the Middle-Income Country

**DOI:** 10.3389/fpubh.2021.634778

**Published:** 2021-03-04

**Authors:** Radoje Simic, Nenad Ratkovic, Viktorija Dragojevic Simic, Zorica Savkovic, Mihajlo Jakovljevic, Vitomir Peric, Milena Pandrc, Nemanja Rancic

**Affiliations:** ^1^Department for Plastic Surgery, Institute for Mother and Child Health Care of Serbia Dr. Vukan Cupic, Belgrade, Serbia; ^2^Faculty of Medicine, University of Belgrade, Belgrade, Serbia; ^3^Medical Faculty of the Military Medical Academy, University of Defence in Belgrade, Belgrade, Serbia; ^4^Sector for Treatment, Military Medical Academy, Belgrade, Serbia; ^5^Center for Clinical Pharmacology, Military Medical Academy, Belgrade, Serbia; ^6^Faculty of Special Education and Rehabilitation, University of Belgrade, Belgrade, Serbia; ^7^Institute of Comparative Economic Studies, Hosei University, Tokyo, Japan; ^8^Department of Global Health Economics and Policy, Faculty of Medical Sciences, University of Kragujevac, Kragujevac, Serbia; ^9^Clinic for Cardiology, Military Medical Academy, Belgrade, Serbia

**Keywords:** cardiovascular diseases, ischemic heart disease, arterial hypertension, military personnel, primary prevention, healthcare costs, middle-income country, Serbia

## Abstract

Cardiovascular diseases, including ischemic heart disease, are the most common causes of morbidity and death in the world, including Serbia, as a middle-income European country. The aim of the study was to determine the costs of preventive examinations for ischemic heart disease in active-duty military personnel, as well as to assess whether this was justified from the point of view of the limited health resources allocated for the treatment of the Republic of Serbia population. This is a retrospective cost-preventive study which included 738 male active-duty military personnel, aged from 23 to 58. The costs of primary prevention of ischemic heart disease in this population were investigated. Out of 738 subjects examined, arterial hypertension was detected in 101 subjects (in 74 of them, arterial hypertension was registered for the first time, while 27 subjects were already subjected to pharmacotherapy for arterial hypertension). Average costs of all services during the periodic-health-examination screening program were €76.96 per subject. However, average costs of all services during the periodic-health-examination screening program for patients with newfound arterial hypertension and poorly regulated arterial hypertension were €767.54 per patient and €2,103.63 per patient, respectively. Since periodic-health-examination screening program in military personnel enabled not only discovery of patient with newfound arterial hypertension but also regular monitoring of those who are already on antihypertensive therapy, significant savings of €690.58 per patient and €2,026.67 per patient can be achieved, respectively. As financial resources for providing health care in Serbia, as a middle-income country, are limited, further efforts should be put on screening programs for ischemic heart disease due to possible significant savings.

## Introduction

Cardiovascular diseases (CVDs), including ischemic heart disease (IHD), are the most common causes of morbidity and death in the world, but with especially high prevalence in low- and middle-income countries (LICs and MICs) (1). Moreover, the Prospective Urban Rural Epidemiology (PURE) study has indicated that this greater mortality exists despite lower burden of CVD risk factors in comparison with high-income countries (HICs), probably as a result of the inferior quality of IHD management and inappropriate control of risk factors in LICs and lower-middle-income countries (LMICs) (2, 3). In addition to a greater care for the social determinants of health and greater investment in health care, far more effective primary prevention is needed (4). This implies more effective strategy against a number of risk factors, such as arterial hypertension (AH), dyslipidemia, diabetes, unhealthy diet, excessive alcohol intake, inadequate salt intake, and psychosocial stress (5). This also applies to the Republic of Serbia, an upper-middle-income country situated in Southeastern Europe, which had a total population of 8,820,000 inhabitants in 2016, with noncommunicable diseases (NCDs) estimated to account for 95% of all deaths (6). Fifty-four percent of deaths were ascribed to CVDs, including IHD, as one of the main public health problems. According to the Institute for Health Metrics and Evaluation data, IHD caused the most deaths in Serbia during the whole period from 2009 to 2019 (7). Moreover, AH as a risk factor was at first place and drove the most death in the same period in Serbia, although it declined to some 12.5% (7). However, AH is the largest one among metabolic factors, which are the predominant risk factors for CVDs, not only in our country but also in the world (8, 9).

Despite earlier beliefs that AH is not significantly prevalent in young people, it was shown that in the population aged between 20 and 40 years, one in eight adult people has high blood pressure (10). Unfortunately, AH already existing at a young age leads to an earlier onset of IHD, stroke, and transient ischemic attacks (11). Although military personnel are usually more fit, has more physical activity and regular physical examinations, and therefore considered to be in lower risk than the rest of the population, it was shown that 13% of the American military staff had AH (12). Therefore, in order to prevent already-mentioned complications both in the civilian population and in active-duty military personnel, health authorities in many countries try to optimize their guidelines for establishing diagnosis and treatment of CVDs, including AH, as well as expenditures connected with these health care activities (13, 14). It was shown, for example, that if a country, such as Nigeria, invests US$2,742 in prevention for each patient, it would be a cost-effective strategy (15). Results were even better for patients with high 10-year CVD risk (15). In the United States, patients with diagnosed AH have US$2,000 more annual costs concerning health care expenditures in comparison to those without AH (16). As far as military population is concerned, AH has important consequences not only on cardiovascular morbidity but also on their ability to perform their regular duties. This population, as well as military authorities, would benefit significantly, taking into account military profession and organization, from a more intensive and detailed survey of cardiovascular risk factors, including blood pressure controls. However, very few studies have addressed this issue. As a result, an extended number of biochemical analyses, as well as specialist examination, were introduced in already-existing health examination screening programs involving active-duty military personnel.

Therefore, the aim of this study was to determine the costs of preventive examinations for IHD in the active-duty military personnel, as well as to assess whether this was justified from the point of view of the limited health resources allocated for the treatment of the Republic of Serbia Army military personnel.

## Methods

### Type of the Study and Patients

This is a retrospective cost-preventive study, performed from September 2018 to July 2019, which included 738 male active-duty military personnel of the Serbian Army, aged from 23 to 58, examined only once during the regular 1-year follow-up.

For the categorization of the level of blood pressure, the average value of all three measurements was used and the information of AH treatment over the last 4 weeks if the person has already taken antihypertensive therapy. According to the 2018 European Society of Cardiology and European Society of Hypertension Guidelines, the examinees were classified according to the values of blood pressure into the following categories: normal blood pressure (systolic blood pressure/diastolic blood pressure: <139/<89 mmHg) and hypertension (systolic blood pressure/diastolic blood pressure: >140 and/or >90 mmHg) (17, 18).

### Study Variables

The costs of primary prevention of IHD of professional military personnel were investigated. This examination included biochemical analyses [complete blood count; urine examination; total cholesterol level; high-density lipoprotein (HDL), low-density lipoprotein (LDL), and triglyceride levels; blood sugar level; blood urea nitrogen and creatinine values; hemoglobin A1c and C-reactive protein test; fibrinogen level; and sedimentation rate], body measurements (body height, body weight, and perimeter of abdomen), blood pressure and pulse rate values, color Doppler echocardiography, and specialist examination, here as preventive procedures.

Data on expenditures were presented as the sum of total costs for all subjects. They were also presented as an average cost per subject, average cost per patient with newfound AH, and average cost per patient with newfound and poorly regulated AH. The criteria for poorly regulated AH were values over 140 and/or 90 mmHg during this preventive examination, although they were already on antihypertensive therapy, according to their medical records.

### Ethical Approval

The principles of ICH Good Clinical Practice were strictly followed, and ethical approval no. 151/2019 (05/11/2019) from the Ethics Committee of the Military Medical Academy was obtained for the study protocol.

This study is part of the research project of the Ministry of Defense of the Republic of Serbia entitled “Primary prevention of IHD of professional military personnel and civilians employed in the Army of the Republic of Serbia.” The aim of this project is to implement modern principles of CVD prevention in the part of the population that is an *ex officio* subject of the regular systematic examinations.

### Statistical Analysis

The data were analyzed using the Statistical Package IBM-SPSS, version 26.0. Categorical variables (subjects with or without AH) were presented as frequency and were analyzed using the chi-square test.

## Results

AH (>140 and/or >90 mmHg) was recorded in 101 subjects out of 738 examined (Table 1). However, out of these 101 subjects, 27 subjects have already been treated for high blood pressure, while 74 examinees were not treated at all. On the other hand, 43 patients with normal blood pressure values (<139 and/or <89 mmHg) were already on antihypertensive medication, while the majority of examinees (594) neither had high blood pressure nor were subjected to pharmacotherapy (Table 1).

**Table 1:** Distribution of male active-duty military personnel examinees subjected to periodic health examination according to both arterial blood pressure values and corresponding pharmacotherapy.

**Arterial hypertension treatment**	**Blood pressure values during the examination**		
	**<139 and/or <89 mmHg**	**>140 and/or >90 mmHg**	***p*-value**
Without	594 (88.92%)	74 (11.08%)	<0.001^*^
With	43 (61.43%)	27 (38.57%)	

* *Chi-square test*.

All expenditures of services performed during the periodic-health-examination screening program which involved 738 male active-duty military personnel in the Republic of Serbia amounted to €56,798.1 (Table 2). The largest part of these costs (63.46% out of total costs) was related to the examination of a medical specialist (35.24%), echocardiography color Doppler examination (21.83%), and complete blood count service (6.39%). The rest of the examinations accounted for 36.54% of all costs, out of which as much as 9.39% accounted for serum cholesterol analysis.

**Table 2:** Expenditures of all services performed during the periodic-health-examination screening programs in active-duty military personnel in the Republic of Serbia.

**Service**	**Total costs (RSD)**	**Total costs (€)**	**% out of total cost**
Complete blood count	428,040	3,627.5	6.39
Urine examination	302,580	2,564.2	4.51
Cholesterol level in serum	124,950	1,058.9	1.86
HDL cholesterol level in serum	219,600	1,861.0	3.28
LDL cholesterol level in serum	123,080	1,043.1	1.84
Triglyceride serum level	161,480	1,368.5	2.41
Blood sugar level	153,090	1,297.4	2.28
Blood urea nitrogen level in serum	139,460	1,181.9	2.08
Creatinine level in serum	191,100	1,619.5	2.85
Hemoglobin A1c test	28,560	242.0	0.43
C-reactive protein test	376,530	3,190.9	5.62
Fibrinogen level	306,340	2,596.1	4.57
Sedimentation rate	79,950	677.5	1.19
Body height measurement	36,650	310.6	0.55
Body weight measurement	51,310	434.8	0.77
Perimeter of abdomen	67,200	569.5	1.00
Blood pressure measurement	73,800	625.4	1.10
Pulse measurement	14,000	118.6	0.21
Echocardiography color Doppler	1,462,860	12,397.1	21.83
Specialist examination	2,361,600	20,013.6	35.24
Total	6,702,180	56,798.1	

*RSD—Serbian dinar*.

The average cost of all services during the periodic-health-examination screening program in active-duty military personnel in the Republic of Serbia (Table 3) was €76.96 per subject. If we take into consideration just patients with newfound AH, the average cost per patient would be €767.54, while if we consider only patients with poorly regulated AH, the average cost per patient would be €2,103.63 per patient. It means that savings per patient for patients with newfound AH and patients with poorly regulated AH would be €690.58 and €2,026.67, respectively (Table 3).

**Table 3:** Average costs of all services and possible savings during the periodic health examination screening program in active-duty military personnel in the Republic of Serbia.

**Average cost**	**Total (RSD)**	**Total (€)**	**Savings per patient (€)**
Per patient (738 patients)	9,081.54	76.96	
Per patient with newfound arterial hypertension (74 patients)	90,570.00	767.54	690.58
Per patient with poorly regulated arterial hypertension (27 patients)	248,228.88	2,103.63	2,026.67

*RSD—Serbian dinar*.

## Discussion

IHD is a leading public health problem in the Republic of Serbia (as well as worldwide), as MIC situated in the Balkan region (1, 7). Large preventive programs are needed for the whole society, and already-determined prevalence and number of ideal cardiovascular health (CVH) metrics in the adult population of Serbia would help in the development of such programs (19). Taking into account this and already-published reports indicating that CVD prevalence rates, as well as prevalence of risk factors associated with CVDs among active-duty army personnel, have increased over the recent years (13, 20), our idea was to examine the possibility of implementation of modern principles of CVD prevention in the part of the population that is an *ex officio* subject of the regular systematic health examinations. In this paper, we report the costs of preventive examinations for IHD in the active-duty military personnel, from the point of view of the limited health resources allocated for the treatment of not only this population but the whole Serbian population as well. In addition to the expanded type of laboratory tests performed, special attention was paid to the values of arterial blood pressure, since AH, as a risk factor for CVDs, was at first place and drove the most death from 2009 to 2019 in Serbia (7). Our results showed that out of 738 active-duty military personnel examinees, AH was detected in 101 subjects. However, in 43 subjects who had normal blood pressure on the periodic examination, it was the result of their everyday treatment. Therefore, it can be concluded that 19.51% (144 out of 738) of the examined military population had AH, both previously diagnosed and newfound ones. Speaking about the whole population, the prevalence of AH among the people aged 20 and older was 33.1% in 2013, in Serbia (26.5% in men and 35.2% in women) (21). According to data from the Republican Health Insurance Fund (RHIF) in Serbia, the number of registered adult patients with AH was 1,477,000, and that was 28.6% of the whole population in 2015 in our country (22, 23). Little is known about AH prevalence in the armed forces (24). In this study involving US military service members, 13% of the 15,391 subjects met the study definition for AH (24). The conclusion of the authors was that the prevalence rates of AH in the US armed forces and the general population were 12.8 and 28%, respectively. However, the high prevalence of prehypertension in comparison to the general male population (62 vs. 47%, respectively) makes it obvious that there is a need to better define the diagnosis and treatment of the prehypertension state in this population. Therefore, AH appears to be a significant problem in the army, too. It has significant impact not only on cardiovascular morbidity but also on the ability of individuals to perform occupational duties. Some aspects of military life may predispose individuals to the development of AH (24). High levels of industrial noise including vehicles, machinery, and weapons systems, as well as aircraft noise, have been linked to it. CVD prevalence rates among US army personnel have increased from 6.8% in 2007 to 9.4% in 2014, as well as the prevalence rates of risk factors associated with CVDs (13, 20). A diagnosis of high blood pressure for, example, was established in 18% of cases, similar to our results (19.51%). Preventive health interventions aimed at blood pressure regulation of service members through physical activity and nutrition may provide significant improvements in cardiovascular health not only for the active military personnel but also in civilians (13, 25).

In addition to the health care status of the military personnel, reduction of the health care costs is also very important for each national army force. In our study, the average cost of all services during the periodic-health-examination screening program was €76.96 per subject. However, the average costs of all services during this program for patients with newfound AH and poorly regulated AH were €767.54 per patient and €2,103.63 per patient, respectively. The economic aspects of AH are very important for the implementation of prevention and therapy of modern principles, in both the civilian and military sectors (26). The high prevalence and costs of the disease impact the gross domestic product (26). Patients with AH incur 2.5 times more inpatient costs, twice the outpatient costs, and triple the prescription medication costs, annually (16). These data derive from a study which included 224,920 adult patients in the United States, of which 83,018 or 36.9% had AH. National medical expenses related to high blood pressure cost more than 3% of the overall health care spending of the United States. Total direct medical costs of CVDs are expected to triple by 2030, when more than 40% of the US population could have some form of CVDs (27, 28). The costs of having AH treated by French general practitioners were estimated, and the average annual cost of treatment was €597 (sum of costs of visits, costs of drugs, and cost of complementary exams) (29). The annual average costs of all drugs (antihypertensive drugs, and other drugs: other cardiovascular drugs, treatment of obesity, diabetes, drugs for tobacco cessation, antifibrinolytic agents, and antiplatelet aggregating agents) were €447, and antihypertensive drugs only accounted for €258. Data from Brazil indicated that direct annual costs of AH treatment were approximately US$398.9 million and US$272.7 million for the Brazilian public health care system and for the Brazilian private health care system, respectively, representing, as a sum, 0.08% of the 2005 gross domestic product (30). According to the Brazilian authors, since available resources are limited, efforts must be focused on the education (screening) of the population on prevention and treatment compliance of AH (30).

Epidemiological burden with chronic NCDs, like CVDs, together with a population which is much older means that difficulties in financing health care will be very serious even for the richest countries, such as nations of the OECD, BRICS, and the Group of Seven (G7) nations (31–34). Difficulties in financing health care are particularly pronounced in developing regions, such as the Balkan, in which Serbia is situated (35, 36). Uneven development and socioeconomic inequalities in health care access are constantly growing. Therefore, Serbian health authorities should focus on AH, among other numerous risk factors of CVDs, and more intensive screening for CVDs is necessary (4, 8, 35–37).

The results of our study enabled us not only to discover the patients with newfound AH but also to regularly monitor those who were already on antihypertensive therapy and detect significant savings of €690.58 per patient and €2,026.67 per patient, respectively. These results encourage us to continue our research, in order to assess the cardiovascular outcomes in our examinees after a longer period of follow-up, as well as the costs not only for prevention but also for the treatment of conditions resulting from numerous risk factors for CVDs in our military personnel. Moreover, 19.51% of the examined military population had AH, while that value in the general population in Serbia accounted for about 27–28% (21–23). However, although it is considered that military personnel are at a lower risk of developing AH, the already-mentioned data indicate that the prevalence rate may become closer to the general population over time. On the other hand, since awareness of the importance of the far more effective primary prevention of CVDs and IHD is rapidly growing in our country (19), new investments of the RHIF are expected, and the funds raised for the services shown in this research should become available to the general Serbian population. There are already positive trends, although modest, since in the 10-year period (from 2009 to 2019), the prevalence rates of IHD and AH were decreased by 2.4 and 12.5%, respectively in the Republic of Serbia, as MIC (7).

The limitation of our study refers to the fact that blood pressure during the periodic health examination of military personnel was registered only once, although the measurements were repeated three times. However, these blood pressure checks in each person have already been performed once a year, in previous years, and will be done regularly in the future. Additional laboratory tests and specialistic examinations were introduced, and costs of preventive examinations for IHD were determined. Moreover, the prices of each service provided are shown, as well as savings achieved as a result of early detection of high blood pressure, as well as of constant monitoring of those individuals who are already on therapy.

## Conclusions

The average cost of all services during the periodic-health-examination screening program for IHD in active-duty military personnel in the Republic of Serbia was €76.96 per subject. However, these examinations enabled not only discovery of the patients with newfound AH but also regular monitoring of those who were already on antihypertensive therapy, and significant savings from €690.58 per patient to even €2,026.67 per patient, respectively, were achieved. Taking into account the very limited financial resources for providing health care in Serbia, as a MIC, in both the civilian and military sectors, further efforts should be put on screening programs for IHD in both populations, due to possible significant savings.

## Data Availability Statement

The original contributions presented in the study are included in the article/supplementary material, further inquiries can be directed to the corresponding author/s.

## Ethics Statement

The studies involving human participants were reviewed and approved by Ethics Committee of the Military Medical Academy. The patients/participants provided their written informed consent to participate in this study.

## Author Contributions

NRat and RS: conceptualization. NRat and NRan: methodology. VP and MP: software. NRat, VDS, ZS, MJ, and NRan: formal analysis. VP, MP, ZS, and NRat: investigation. RS, NRat, VDS, and NRan: writing—original draft preparation. RS, NRat, VDS, MJ, and NRan: writing—review and editing. NRat: project administration. All authors have read and agreed to the published version of the manuscript.

## Conflict of Interest

The authors declare that the research was conducted in the absence of any commercial or financial relationships that could be construed as a potential conflict of interest.
